# Test–retest reliability of cortico-spinal measurements in the rectus femoris at different contraction levels

**DOI:** 10.3389/fnins.2023.1239982

**Published:** 2023-10-02

**Authors:** Gonzalo Gomez-Guerrero, Janne Avela, Miro Enroth, Ella Häkkinen, Paul Ansdell, Glyn Howatson, Simon Walker

**Affiliations:** ^1^Faculty of Sport and Health Sciences, NeuroMuscular Research Center, University of Jyväskylä, Jyväskylä, Finland; ^2^Faculty of Health and Life Science, Northumbria University, Newcastle upon Tyne, United Kingdom; ^3^Water Research Group, North West University, Potchefstroom, South Africa

**Keywords:** reliability, lumbar stimulation, spinal excitability, silent period, cortico-spinal tract, lower limb, knee extensors

## Abstract

Single-pulse Transcranial Magnetic Stimulation (TMS) and, very recently, lumbar stimulation (LS) have been used to measure cortico-spinal excitability from various interventions using maximal or submaximal contractions in the lower limbs. However, reliability studies have overlooked a wide range of contraction intensities for MEPs, and no reliability data is available for LEPs. This study investigated the reliability of motor evoked potentials and lumbar evoked potentials at different stimulation intensities and contraction levels in m.rectus femoris. Twenty-two participants performed non-fatiguing isometric knee extensions at 20 and 60% of maximum voluntary contraction (MVC). LS induced a lumbar-evoked potential (LEP) of 25 and 50% resting maximal compound action potential (M-max). TMS stimulator output was adjusted to 120, 140, and 160% of active motor threshold (aMT). In each contraction, a single MEP or LEP was delivered. Ten contractions were performed at each stimulator intensity and contraction level in random order. Moderate-to-good reliability was found when LEP was normalized to M-max/Root Mean Square in all conditions (ICC:0.74–0.85). Excellent reliability was found when MEP was normalized to Mmax for all conditions (ICC > 0.90) at 60% of MVC. Good reliability was found for the rest of the TMS conditions. Moderate-to-good reliability was found for silent period (SP) elicited by LS (ICC: 0.71–0.83). Good-to-excellent reliability was found for SP elicited by TMS (ICC > 0.82). MEPs and LEPs elicited in m.rectus femoris appear to be reliable to assess changes at different segments of the cortico-spinal tract during different contraction levels and stimulator output intensities. Furthermore, the TMS- and LS- elicited SP was a reliable tool considered to reflect inhibitory processes at spinal and cortical levels.

## Introduction

Transcranial magnetic stimulation (TMS) is a safe and non-invasive technique used over the skull to elicit a response in a specific target. A single-pulse stimulus over the contralateral motor cortex of a specific muscle will induce a descending volley, by transynaptically activating pyramidal cells, creating a muscle action potential recorded by electromyography (EMG) in the muscle targeted (Barker et al., 1985). The action potential generated by TMS is known as a motor evoked potential (MEP) and changes in its size provides information about cortico-spinal excitability (Day et al., 1989; Kobayashi and Pascual-Leone, 2003). However, MEPs cannot dissociate between changes at cortical or spinal level excitability (Taylor, 2006). Dissociating between cortical and spinal excitability changes could lead to a better understanding of the nervous system and how different segments of the nervous system respond to different interventions (Butler et al., 2003; Taylor, 2006; McNeil et al., 2009). Therefore, other methodologies, using transcutaneous electrical stimulation, have been validated to assess spinal excitability (Ugawa et al., 1991; Petersen et al., 2002; Taylor et al., 2002; Martin et al., 2008; Škarabot et al., 2019b).

Transcutaneous electrical stimulation at the spinal level has been used in the literature to assess spinal excitability at different levels (Ugawa et al., 1995; Gandevia et al., 1999; Škarabot et al., 2019a; Brownstein et al., 2020). Cervicomedullary stimulation has been used to elicit a monosynaptic response in upper and lower limb muscles through activation of cortico-spinal tract neurons. The induced action potential is known as a cervicomedullary motor evoked potential (CMEP). However, high intensities are required to elicit a CMEP in the lower limbs, which may be only visible in some participants (Ugawa et al., 1991, 1995). Given the importance of understanding the neural mechanisms in the lower limbs for health and performance, some authors have performed spinal stimulations to other segments of the spine (Martin et al., 2008; Škarabot et al., 2019b). Martin et al. (2008) and Škarabot et al. (2019b) validated thoracic stimulation and lumbar stimulation, respectively, by demonstrating that both stimulations can activate axons of the cortico-spinal tract without activating ventral or dorsal roots. The action potential elicited by these stimulations are known as a thoracic motor evoked potential (TMEP) and a lumbar evoked potential (LEP) (Martin et al., 2008; Škarabot et al., 2019b). Nevertheless, the site of thoracic stimulation (T3 and T4) is far from lower limb motor-neurons (L3–L5) as opposed to lumbar stimulation, where the center of stimulation is approximately at L1 (Martin et al., 2008; Škarabot et al., 2019b). This difference leads to higher stimulation intensities in thoracic than lumbar stimulation, which makes the thoracic method more unpleasant (Brownstein et al., 2020), similar to cervicomedullary stimulations. Thus, lumbar stimulation may be considered a more appropriate method to target the lower limbs. In addition the silent period (SP), considered as an interruption of the EMG during voluntary muscle contraction after TMS, can also be observed after lumbar stimulation (LS), and has been reported as a measure of inhibition at the spine (Merton, 1951; Inghilleri et al., 1993). Despite validation studies and the increased use of these methodologies in clinical and sport science settings, there is a lack of reliability studies examining both LEP amplitude and its SP.

Further, even though a number of studies have reported MEP reliability (O’Leary et al., 2015; Beaulieu et al., 2017; Peri et al., 2017; Temesi et al., 2017; Houde et al., 2018; Leung et al., 2018), a limited number of studies have examined reliability during maximal (Malcolm et al., 2021) or submaximal contractions in the knee extensors (at 10% of MVC: O’Leary et al., 2015; Brownstein et al., 2018; Leung et al., 2018; Pagan et al., 2023; and at 20% of MVC: Temesi et al., 2017). Interestingly, MEPs showed good-to-excellent (ICC: 0.78–0.90) reliability during low submaximal contractions (10–20% of MVC) (O’Leary et al., 2015; Temesi et al., 2017; Brownstein et al., 2018), but poor reliability (ICC = 0.56) was found during maximal contractions intensities (100% of MVC) (Malcolm et al., 2021). Moreover, MEPs and CMEPs increased similarly during a sustained task at 50% of MVC on the biceps brachii (Lévénez et al., 2008), whereas MEPs increased to a greater extent than CMEPs during a 30% of MVC in the plantar flexors (Hoffman et al., 2009). Such findings demonstrate the possible impact of various contraction intensities on electrophysiological data. In addition, strength training and acute fatigue studies have used a wide range of contraction intensities (20–100% of MVC) to assess cortical and/or spinal excitability (Butler et al., 2003; Lévénez et al., 2008; Goodall et al., 2009; Hoffman et al., 2009; Sidhu et al., 2009; Tallent et al., 2017), for which prior reliability has not been established. Thus, there is a need to determine reliability of MEP and LEP data from lower limbs to allow full evaluation of previous and future intervention studies.

Examining the contributing factors of cortical or spinal excitability in the locomotor muscles is important for determining exercise-induced alterations in nervous system function throughout the spectrum of health, exercise and disease (Sidhu et al., 2013). Furthermore, m.rectus femoris (RF) is involved in lower limb swing actions and stability (Landin et al., 2016), playing an important role in locomotion. Therefore, the aim of this study is to assess the test–retest reliability of MEP and LEP at different stimulator output intensities and different submaximal contraction levels in RF in a wide age range of asymptomatic adults.

## Materials and methods

### Participants

Twenty-seven participants volunteered for the study (14 female). Five participants were removed during the offline analysis due to possible activation of ventral roots (see Lumbar-evoked potentials). Therefore, the data presented here are representative of the 22 (12 female) volunteers fulfilling all study requirements (47 ± 23 years; height: 171 ± 10 cm; body mass: 80 ± 20 kg). All included participants were free from neurological illness and musculoskeletal injury in the lower-limbs for the last 6 months, were not taking any medications known to affect the nervous system and had no contraindications to transcranial magnetic stimulation (TMS), which was assessed via a health questionnaire (modified from Rossi et al., 2011). Before testing, all participants were fully informed of the procedures and possible risks, and each participant provided written inform consent. The Ethical committee of the University of Jyväskylä provided a statement for the study (857/13.00.04.00/2021) and the study was conducted in accordance with the ethical standards establish in the *Declaration of Helsinki* (2013).

### Experimental set-up

Participants visited the laboratory on five occasions. The first session was a familiarization session, where the participants were introduced to all instructions and stimulations that were given during the testing sessions. Furthermore, this session was used for preliminary assessment of the lumbar stimulation electrode placement and transcranial magnetic stimulation intensity for motor threshold determination. The other four sessions were testing sessions: two of them were dedicated for Lumbar Stimulation (LS) and the other two for TMS stimulation. One session of each stimulation method was performed 10–14 days prior to the second one. For each participant, sessions were performed at the same time of day. TMS was performed at least 48 h after LS.

**Figure 1:**
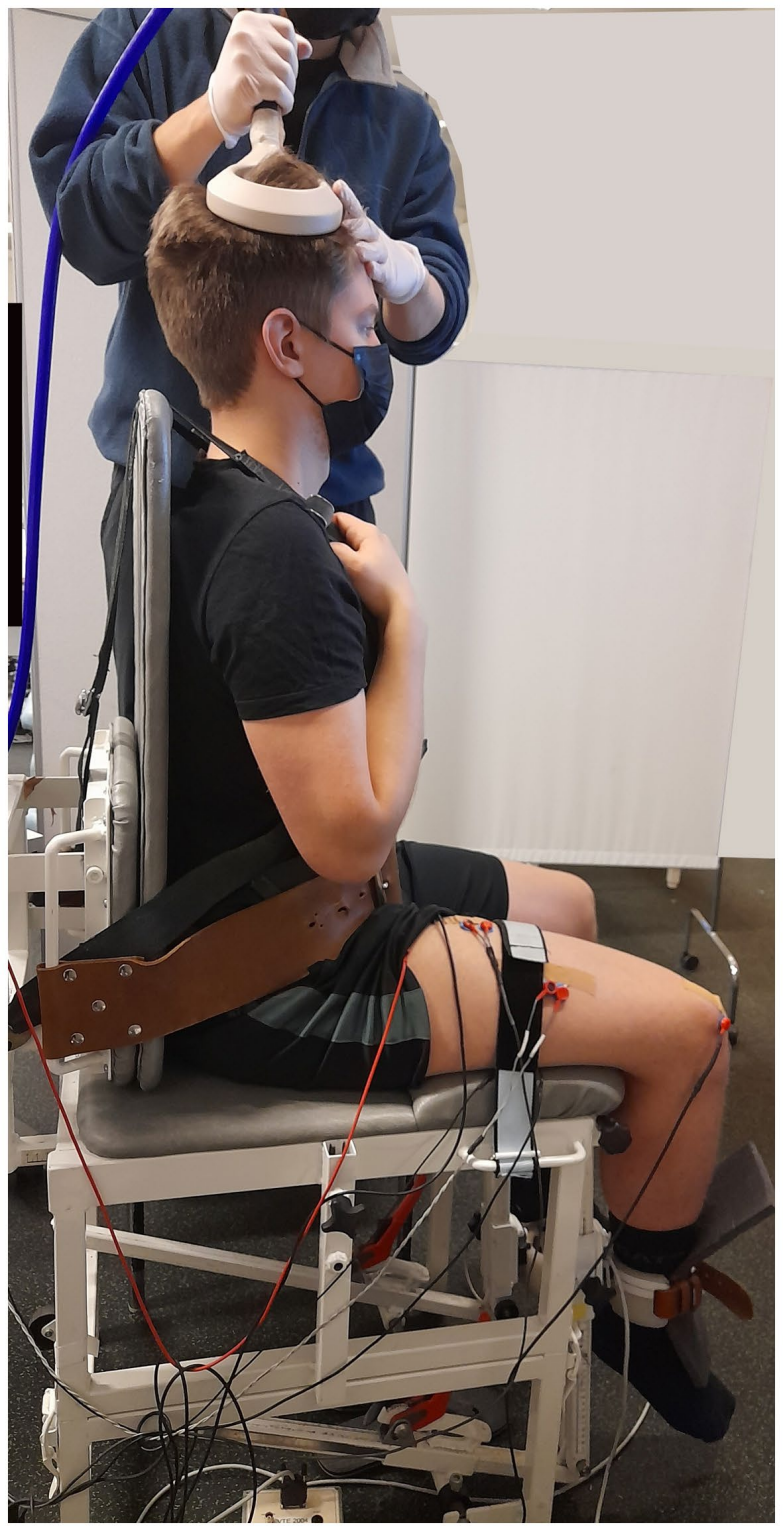
Representation of participants set-up during the testing session.

To assess responses in RF, participants sat in a custom-built chair with a calibrated load cell (Faculty of Sport and Health Sciences, University of Jyväskylä, Finland) with hip and knee at 90° flexion and the shin strapped by a non-elastic restraint ~2 cm superior to the ankle malleoli (Figure 1). The voltage signal originating from the load cell was calibrated and converted into torque (N·m). All measures were performed on the right (i.e., dominant) leg.

Every session followed the same structure. Once the participant was secured to the dynamometer, the maximum compound action potential (M-max) was assessed in a relaxed condition (i.e., M-maxpre). Two maximal voluntary contraction (MVC) trials were performed 60 s apart (i.e., MVCpre). Prior to the MVC, two contractions at ~50% and ~ 80% of estimated MVC were performed as a warm-up. Verbal encouragement and visual feedback were provided to motivate participants to produce maximal effort and torque was recorded.

**Figure 2:**
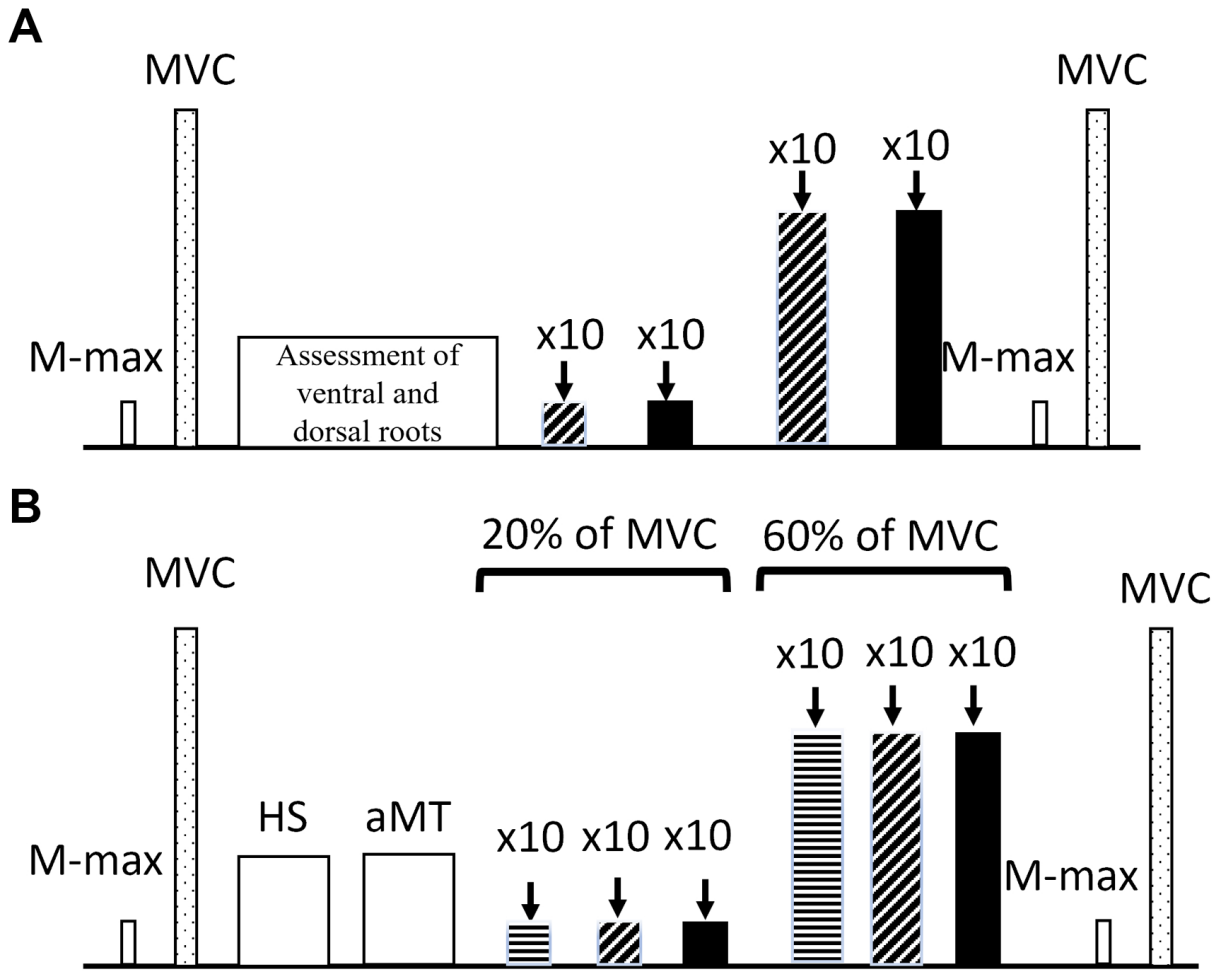
Representation of **(A)** lumbar session settings, low intensity (diagonal stripes) and high intensity (filled up bars). and **(B)** TMS session settings with 120% aMT (horizontal stripes), 140% aMT (diagonal stripes) and 160% aMT (filled bars). M-max: maximal compound action potential, MVC: Maximal voluntary contraction, HS, hotspot; aMT, active motor threshold.

In every testing session, visual feedback was provided to the participants to produce the required submaximal torque and then a single TMS/LS stimulus was delivered manually. Contractions at 20 and 60% of MVC were held for 5–8 s. Sets of 10 stimulations were given per condition and per contraction level as a single block, giving a total of 40 LS and 60 TMS stimulations. To avoid fatigue, 30 s and 45 s rest was given between contractions during 20 and 60% of MVC, respectively, and 60 s and 180 s rest was given between the sets of 10 contractions. At the end of the protocol, M-max (M-maxpost) and MVC (MVCpost) were re-assessed (Figure 2).

### Peripheral nerve stimulation

Transcutaneous electrical stimulation of the femoral nerve (32 mm cathode/anode arrangement; Polar Neurostimulation Electrodes, Espoo, Finland) was performed to elicit M-max in RF (1 ms pulse duration; Digitimer DS7AH, Hertfordshire, United Kingdom). Electrodes were placed 2 cm apart and placed at each side of the femoral nerve, located by palpation and identification of the femoral artery (Walker et al., 2016). M-max was elicited by gradually increasing stimulator output intensity until the EMG response plateaued. To ensure supramaximality, this intensity was further increased by 50% (Table 1).

**Table 1:** Mean and standard deviation values of torque, peripheral nerve stimulation, TMS and LS parameters from the participants during test sessions 1 and 2.

		Test 1	SD	Test 2	SD	Value of *p*	95% CI	Hedges’ *g*
	MVC (n.m)	175	60	179	62	0.286	[−10, 3]	−0.06
Peripheral								
	Peripheral							
	M-max (mV)	1.99	1.17	1.91	0.88	0.694	[−0.17, 0.30]	0.08
	Peripheral Stim. intensity (mA)	220	93	229	79	0.698	[−61, 42]	−0.10
TMS								
	aMT (%)	35	9	35	8	0.555	[−2, 1]	0.00
LS								
	25% M-max Stim. intensity (mA)	240	98	231	92	0.577	[−18, 31]	0.09
	LEP onset 25% M-max latency (ms)	8.5	1.4	8.5	1.8	0.685	[−3.5, 5.2]	0.00
	50% M-max Stim. intensity (mA)	274	104	273	104	0.958	[−36, 34]	0.01
	LEP onset 50% M-max latencies (ms)	8.4	1.6	8.3	1.8	0.647	[−3.5, 5.5]	0.06

SD, standard deviation; CI, confidence interval; MVC, maximal voluntary contraction; TMS, transcranial magnetic stimulation; LS, lumbar stimulation; M-max, maximal compound action potential; aMT, active motor threshold; LEP, lumbar evoked potential; Stim., stimulation.

### Transcranial magnetic stimulation

Single TMS pulses were delivered using a Magstim 200^2^ magnetic stimulator (Magstim Co., Ltd., Whitland, United Kingdom) connected to a concave double-cone coil, positioned over the left cortical hemisphere for RF with a posterior-to-anterior current orientation, to elicit MEPs. The hotspot was defined, at rest, as the position eliciting the largest visible MEP recorded in the EMG using the same intensity (approx. 50–70% stimulator output). Once the hotspot was found, the coil position was marked on the scalp to maintain the same position throughout the protocol. Active motor threshold (aMT) was determined by increasing stimulator intensity in 5% steps, starting at 30% of the stimulator output. Thereafter, stimulator intensity was decreased in steps of 1% until clear MEPs (>100 μV) were elicited in 3 out of 5 stimulations during 10% of MVC. Sets of 10 single TMS stimulations were delivered in a random order for each of the assigned conditions (i.e., 120, 140, and 160% aMT).

### Lumbar stimulation

Transcutaneous electrical lumbar stimulation was used to elicit LEPs with a constant-current stimulator (1 ms pulse duration; Digitimer DS7AH, Hertfordshire, United Kingdom) via self-adhesive electrodes (Polar Neurostimulation Electrodes, Espoo, Finland). The cathode (5 × 10 cm) was centered over the first lumbar vertebra (L_1_) and the anode (circular shape; 5 cm diameter) was placed on the midline of the vertebral column ~5 cm above the top edge of the cathode as described by Škarabot et al. (2019a).

The intensity of stimulation was standardized to 25% or 50% of the M-max evoked in the resting position (Table 1). Potential activation of ventral roots was assessed by examining the onset latency of the LEP with an increase in stimulator intensity (Petersen et al., 2002) and tracking LEP amplitude during increased voluntary contraction (Taylor et al., 2002). Onset latency would be expected to shorten when increasing stimulation intensity and LEP amplitude would have remained consistent during higher contraction intensities should the ventral roots be activated (Petersen et al., 2002; Taylor et al., 2002, 2006; Škarabot et al., 2019b). To ensure the placement was the same in both sessions, the distance from the 7^th^ cervical vertebra (C7) to the anode and from the bottom of the anode to the top of the cathode (i.e., inter-electrode distance) were taken. Five out of the 27 participants showed no increase in LEP amplitude with an increase in voluntary torque, and they were, therefore, removed from further analyses.

**Figure 3:**
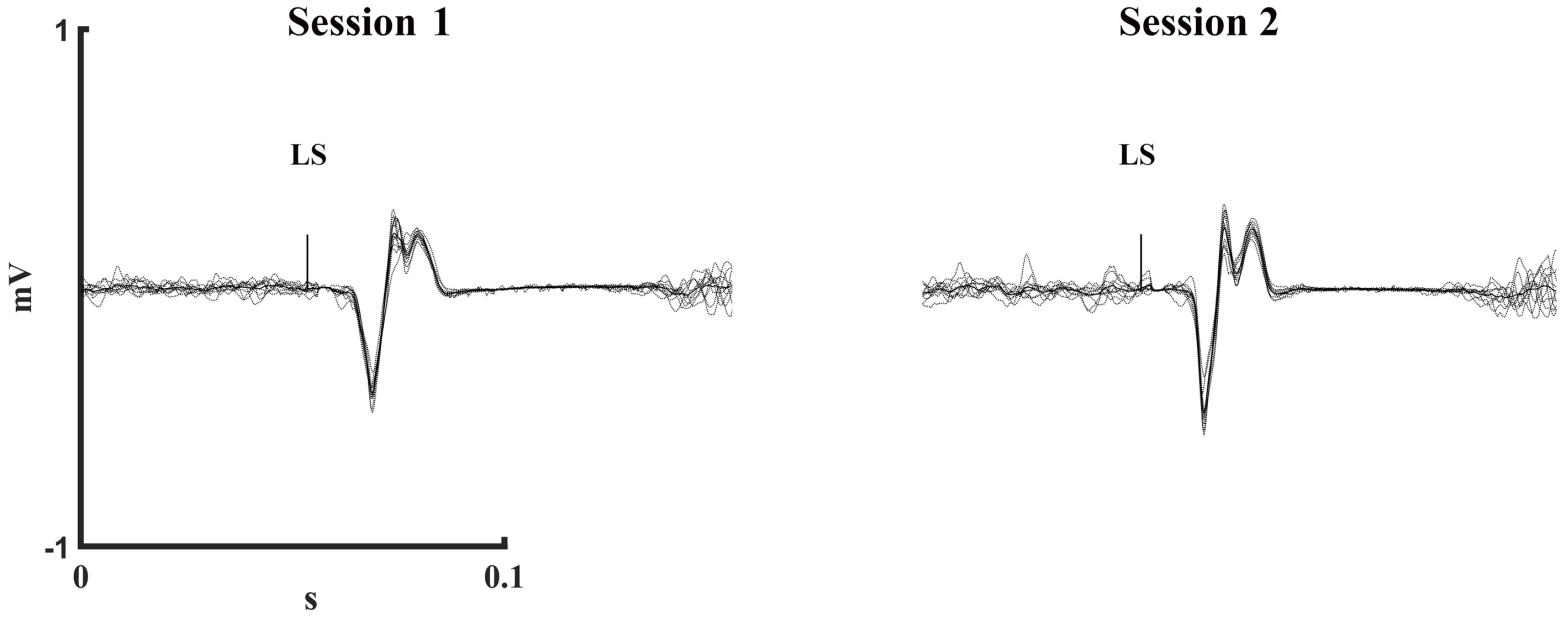
One participant’s mean (solid) and individual (dashed) trials of low intensity LEPs at 20% of MVC during test session 1 and 2. LS, Lumbar stimulation.

Dorsal root activation was assessed via paired LS with a 50 ms time delay, where the second LEP amplitude was compared to the first. Evidence of dorsal root activation would be a decrease in the second LEP compared to the first due to post-activation depression at the motor-neuron pool from the first stimulus to the second (Hofstoetter et al., 2018). All remaining participants showed no sign of the responses described and reported that they found LS to be tolerable. Once the placement was confirmed, stimulator intensity was adjusted to that which produced a LEP of 25% (Low intensity) or 50% (High intensity) of the M-max at rest, and these stimulation intensities were used throughout the experiment (Figure 3).

### Bipolar surface electromyography and torque

Muscle activity was recorded using adhesive Ag/AgCl electrodes (30 × 20mm, BlueSensor N, Ambu, Penang, Malaysia) from m.Bicep Femoris (BF) and RF according to SENIAM guidelines (Hermens et al., 2000). Skin was shaved, abraded with sandpaper, and wiped with alcohol before setting the electrodes in bipolar arrangement with 2 cm center-to-center distance. Impedance was set <2 kΩ, and the reference electrode was positioned above the patella. EMG data were sampled online at 3000 Hz, amplified (1000×) and bandpass filtered (16–1,000 Hz; Neurolog System, Digitimer Ltd., United Kingdom) using CED Power1401-3 (Cambridge Electronic Design Ltd., Cambridge, United Kingdom).

Torque was sampled at 1000 Hz, amplified by a custom-built amplifier (ForAmps 1 v1.2, University of Jyväskylä, Finland) and converted by a 16-bit A/D board (CED Power1401-3, Cambridge Electronics Design, Cambridge, United Kingdom) in combination with Spike2 software (version 6.10, Cambridge Electronic Design, Cambridge, United Kingdom).

### Data and statistical analyses

Offline analyses were performed with Spike2 software (version 6.10, Cambridge Electronic Design, Cambridge, United Kingdom) to manually obtain M-max amplitude, MVC, and unconditioned LEP onset latencies. The other outcome measures were analyzed by a customized MATLAB script (version R2020b, The MathWorks, Inc., Natick, United States). Peak-to-peak amplitude of MEPs and LEPs were analyzed automatically between latencies-of-interest following TMS or LS (Taylor et al., 1999), respectively. Silent period and onset latencies were analyzed semi-automatically, by visually selecting the end of the SP and the onset for MEPs and LEPs. The median values for each set of 10 MEPs, LEPS, MEP SPs and LEP SPs were calculated, as the median is less sensitive to outliers. Torque and Root Mean Square (RMS) of the EMG were averaged over the 100 ms before the stimulator artefact (Škarabot et al., 2019c). SP duration was determined, through visual inspection, as the time from the stimulator artefact to the return of voluntary EMG (Damron et al., 2008). Normalization of MEP and LEP amplitude was performed by normalizing to M-max (Single *N*) or to M-max and then RMS (Double *N*). Double *N* is typically performed to avoid the possibility that the background EMG level might modify the MEP or LEP (Sidhu et al., 2013; Škarabot et al., 2019a).

SPSS software (version 26.0, SPSS Inc., Chicago, United States) was used for all statistical methods. Means and standard deviation (SD) were calculated and reported throughout. Normality of the data was tested with the Shapiro–Wilk test and confirmed by z-score with an acceptance of +2 to −2 (e.g., skewness score/skewness score_SE_ and kurtosis score/kurtosis score_SE_). Data that did not fulfil those requirements: 20% of MVC Single N at low intensity, 120% aMT and 160% aMT; double N at low intensity, high intensity, 120% aMT, 140% aMT and 160% aMT; SP at low intensity, 140% aMT and 160% aMT; 60% of MVC SP at 160% aMT were Log10 transformed, which then fulfilled the requirements for normality. Paired *t*-tests were used to examine difference between means trials of Single N and Double N for MEP and LEP amplitude and SP. Statistical significance was accepted at alpha <0.05. Between-group effect sizes are represented as Hedge’s *g* for the relative changes over time (*g* = small: <0.3, medium: 0.3–0.8, large: >0.8). Relative reliability, as the degree to which individuals maintain their position in a sample with repeated measurements, of TMS and LS were assessed using Intraclass Correlation coefficient (ICC). Absolute reliability, as the degree to which repeated measurements vary within individuals, was assessed using typical error (TE), coefficient of variance (CV) and standard error of the measurement (SEM) calculated as: $\mathrm{averaged}\;SD\;\mathrm{of}\;\mathrm{test}\;1\;\mathrm{and}\;\mathrm{test}\;2\;\times \;\sqrt{1-ICC}$ (Portney, 2020) expressed in ratio (Single N or Double N) or time (SP) for MEPs and LEPs (Atkinson and Nevill, 1998; Portney, 2020). The minimal detectable change (MDC) was calculated as SEM × 1.96 × $\sqrt{2}$ . Reliability, based on ICCs and their 95% CIs, was categorized as poor (ICC < 0.5), moderate (ICC: > 0.5 - < 0.75), good (ICC: > 0.75 - < 0.9) and excellent (ICC: > 0.9) (Koo and Li, 2016). Bland–Altman plots of LEPs and MEPs in all conditions were used to assess the agreement between the two sessions.

## Results

### Control measurements

As shown in Table 1, stimulation variables that could potentially influence changes at cortical and spinal levels were assessed for potential differences between session 1 and session 2. None of the variables assessed were statistically significant and, thus, were stable from one test session to the next.

### Reliability of lumbar evoked potentials at different submaximal contraction levels

**Figure 4:**
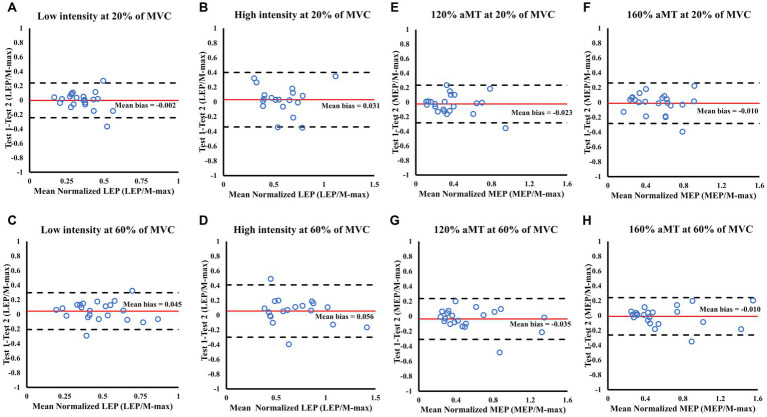
Bland Altman plots showing the level of agreement for LEP **(A–D)** and MEP **(E–H)** amplitude during 20 and 60% of MVC between test sessions 1 and 2. Each panel shows the differences as a function of the average of the two testing sessions indicating the mean bias (solid line) and the 95% limits of agreement (dashed lines).

There were no significant changes in LEP amplitude elicited at any intensity from test 1 to test 2, regardless of whether Single *N* or Double *N* was used, at any contraction level (Table 2). All reliability values for LS can be found in Table 3. Good reliability was found in Double N for LEPs elicited at high intensity during 20% of MVC (ICC = 0.847) and at low intensity during 60% of MVC (ICC = 0.828), while moderate reliability was found for the rest of the conditions (Table 3). CVs for Single N was 23% for lower intensities, independent of the contraction level, whereas at high intensities CVs of 29 and 33% were observed. SEM for Single *N* was between 0.07–0.17 and MDC was between 0.20–0.47. CV for Double N was between 30–39%. SEM was between 2–10 and MDC was between 6–27. Bland–Altman plots showed a good agreement between test 1 and test 2 for all LS conditions (Figures 4A–D). Low intensity stimulation during 20% of MVC showed a mean bias of −0.002 and 95% limits of agreement [−0.24, 0.24] (Figure 4A).

**Table 2:** Mean and standard deviation, 95% confidence intervals, effect sizes and results of paired *t*-test analyses for Single *N* and Double *N* LEP amplitudes and LEP SP comparisons between test sessions 1 and 2.

			Test 1	SD	Test 2	SD	value of p	95% CI	Hedges’ *g*
20% of MVC									
	Low intensity								
	Single *N* (LEP/M-max)		0.352	0.106	0.354	0.135	0.95	[−0.056, 0.053]	−0.02
	Double *N* (LEP/M-max/RMS)		19.1	13.7	17.8	13.1	0.45	[−2.1, 4.6]	0.09
	Silent period (ms)		79	12	77	12	0.26	[−2, 6]	0.16
	High intensity								
	Single *N* (LEP/M-max)		0.597	0.219	0.566	0.226	0.48	[−0.060, 0.122]	0.14
	Double *N* (LEP/M-max/RMS)		29.0	22.5	29.0	27.2	0.99	[−4.2, 4.3]	0.00
	Silent period (ms)		87	15	86	12	0.62	[−3, 5]	0.07
60% of MVC									
	Low intensity								
	Single *N* (LEP/M-max)		0.51	0.184	0.454	0.189	0.115	[−0.012, 0.102]	0.29
	Double *N* (LEP/M-max/RMS)		7.9	6.2	7.0	4.8	0.110	[−0.3, 2.6]	0.15
	Silent period (ms)		69	14	67	16	0.348	[−2, 7]	0.13
	High intensity								
	Single *N* (LEP/M-max)		0.717	0.26	0.655	0.311	0.193	[−0.031, 0.143]	0.21
	Double *N* (LEP/M-max/RMS)		11.4	9.2	10.1	6.7	0.300	[−1.8, 5.5]	0.16
	Silent period (ms)		68	10	67	9	0.528	[−2, 4]	0.10

SD, standard deviation; CI, confidence interval; LS, lumbar stimulation; LEP, lumbar evoked potential; M-max, maximal compound action potential; RMS, root mean square.

**Table 3:** Between-session test–retest reliability for Single *N* and Double *N* LEP amplitudes and LEP SP with ICC, TE, SEM, and MDC.

			TE [95%CI]		CV% [95% CI]		ICC [95% CI]		SEM	MDC
20% of MVC										
	Low intensity									
	Single *N* (LEP/M-max)		0.09	[0.07–0.12]	23.0	[17.3–34.5]	0.632	[0.29–0.83]	0.07	0.20
	Double *N* (LEP/M-max/RMS)		5.3	[4.1–7.7]	38.5	[28.5–59.3]	0.737	[0.46–0.88]	6.86	19.02
	Silent period (ms)		5.9	[4.6–8.5]	7.5	[5.7–10.9]	0.713	[0.42–0.87]	6.43	17.82
	High intensity									
	Single *N* (LEP/M-max)		0.13	[0.10–0.20]	33.4	[24.3–53.2]	0.520	[0.09–0.79]	0.15	0.43
	Double *N* (LEP/M-max/RMS)		6.3	[4.7–9.3]	30.0	[22.0–47.5]	0.847	[0.64–0.94]	9.71	26.90
	Silent period (ms)		5.4	[4.1–8.1]	6.7	[5.0–10.0]	0.830	[0.60–0.93]	5.57	15.43
60% of MVC										
	Low intensity									
	Single *N* (LEP/M-max)		0.09	[0.07–0.13]	22.8	[17.1–34.1]	0.749	[0.48–0.89]	0.09	0.26
	Double *N* (LEP/M-max/RMS)		2.3	[1.8–3.3]	31.9	[23.8–48.5]	0.828	[0.62–0.93]	2.27	6.30
	Silent period (ms)		7.3	[5.7–10.5]	11.8	[9.0–17.3]	0.710	[0.41–0.87]	8.08	22.39
	High intensity									
	Single *N* (LEP/M-max)		0.13	[0.10–0.19]	28.8	[21.1–45.4]	0.643	[0.24–0.82]	0.17	0.47
	Double *N* (LEP/M-max/RMS)		5.4	[4.1–7.9]	39.5	[28.6–63.7]	0.742	[0.43–0.90]	4.04	11.20
	Silent period (ms)		5.1	[3.9–7.6]	7.9	[5.9–11.9]	0.750	[0.44–0.90]	4.75	13.17

TE, typical error; CI, confidence interval; CV, coefficient of variance; ICC, intra-class correlation; SEM, standard error of the measurement; MDC, minimal detectable change; MVC, maximal voluntary contraction; LS, lumbar stimulation; LEP, lumbar evoked potential; M-max, maximal compound action potential; RMS, root mean square.

### Reliability of motor evoked potentials at different contraction levels

MEP amplitude elicited at all intensities did not show any changes (*p* > 0.05) from test 1 to test 2, regardless of which normalization or contraction level was used (Table 4). Excellent reliability was found in Single N for all the TMS conditions (ICC > 0.900) and at 140% aMT for Double N (ICC = 0.926) at 60% of MVC. Good reliability was found for the rest of the conditions (Table 5). CVs for Single N was between 20–26% during 20% of MVC, whereas 60% of MVC showed CVs of 14–18%. SEM for Single N was between 0.09–0.13 and MDC was between 0.24–0.36. CV for Double N was between 29–35% for lower contraction levels. SEM was between 6–9 and MDC was between 6–25. Bland–Altman plots showed good agreement between test 1 and 2, with a low ratio (MEP/M-max) for the mean bias (−0.010) and data within the 95% limits of agreement (Figures 4E–H).

**Table 4:** Mean and standard deviation, 95% confidence intervals, effect sizes and results of paired *t*-test analyses for Single *N* and Double *N* MEP amplitudes and MEP SP comparisons between test sessions 1 and 2.

			Test 1	SD	Test 2	SD	value of p	95% CI	Hedges’ *g*
20% of MVC									
	120% aMT								
	Single *N* (MEP/M-max)		0.366	0.223	0.389	0.48	0.421	[−0.082, 0.035]	−0.06
	Double *N* (MEP/M-max/RMS)		19.0	19.4	18.1	10.5	0.656	[−3.4, 5.3]	0.06
	Silent period (ms)		107	19	112	16	0.031	[−9, −1]	−0.28
	140% aMT								
	Single *N* (MEP/M-max)		0.472	0.195	0.479	0.224	0.812	[−0.062, 0.049]	−0.03
	Double *N* (MEP/M-max/RMS)		29.6	30.2	27.0	23.3	0.405	[−3.7, 8.7]	0.09
	Silent period (ms)		127	27	131	27	0.106	[−11, 1]	−0.15
	160% aMT								
	Single *N* (MEP/M-max)		0.509	0.224	0.519	0.240	0.742	[−0.072, 0.052]	−0.04
	Double *N* (MEP/M-max/RMS)		28.0	25.2	26.9	21.0	0.653	[−3.9, 6.2]	0.05
	Silent period (ms)		143	31	144	28	0.468	[−7, 3]	−0.03
60% of MVC									
	120% aMT								
	Single *N* (MEP/M-max)		0.54	0.317	0.558	0.351	0.246	[−0.097, 0.026]	−0.05
	Double *N* (MEP/M-max/RMS)		9.4	9.3	8.8	7.3	0.520	[−1.4, 2.7]	0.08
	Silent period (ms)		106	22	107	17	0.463	[−7, 3]	−0.05
	140% aMT								
	Single *N* (MEP/M-max)		0.552	0.321	0.582	0.366	0.399	[−0.101, 0.042]	−0.09
	Double *N* (MEP/M-max/RMS)		9.3	8.3	10.0	8.8	0.442	[−2.3–1.1]	−0.07
	Silent period (ms)		122	23	124	19	0.245	[−8, 2]	−0.09
	160% aMT								
	Single *N* (MEP/M-max)		0.587	0.368	0.597	0.372	0.723	[−0.067, 0.047]	−0.03
	Double *N* (MEP/M-max/RMS)		10.0	9.1	9.8	8.2	0.810	[−1.7, 2.1]	0.03
	Silent period (ms)		140	35	145	34	0.081	[−10, 1]	−0.14

SD, standard deviation; CI, confidence interval; TMS, transcranial magnetic stimulation; M-max, maximal compound action potential; RMS, root mean square; aMT, active motor threshold; MEP, motor evoked potential.

**Table 5:** Between-session test–retest reliability for Single *N* and Double *N* MEP amplitudes and MEPSP with ICC, TE, SEM, and MDC.

			TE [95%CI]		CV% [95% CI]		ICC [95% CI]		SEM	MDC
20% of MVC										
	120% aMT									
	Single *N* (MEP/M-max)		0.09	[0.07–0.13]	26.1	[19.5–39.3]	0.861	[0.69–0.94]	0.13	0.36
	Double *N* (MEP/M-max/RMS)		7.0	[5.4–10.0]	34.8	[25.9–53.3]	0.816	[0.60–0.92]	6.40	17.74
	Silent period (ms)		7.2	[5.5–10.3]	7.2	[5.5–10.4]	0.820	[0.61–0.92]	7.42	20.58
	140% aMT									
	Single *N* (MEP/M-max)		0.09	[0.07–0.13]	20.6	[15.5–30.7]	0.831	[0.63–0.93]	0.09	0.24
	Double *N* (MEP/M-max/RMS)		9.9	[7.6–14.2]	29.2	[21.8–44.3]	0.891	[0.75–0.95]	8.83	24.47
	Silent period (ms)		9.3	[7.2–13.3]	7.9	[6.0–11.5]	0.840	[0.64–0.93]	10.80	29.94
	160% aMT									
	Single *N* (MEP/M-max)		0.10	[0.08–0.14]	24.0	[17.9–35.9]	0.821	[0.61–0.92]	0.10	0.27
	Double *N* (MEP/M-max/RMS)		8.1	[6.2–11.5]	29.9	[22.3–45.3]	0.851	[0.67–0.94]	8.93	24.74
	Silent period (ms)		7.7	[6.0–11.1]	5.5	[4.2–8.0]	0.920	[0.81–0.97]	8.34	23.13
60% of MVC										
	120% aMT									
	Single *N* (MEP/M-max)		0.1	[0.08–0.14]	18.4	[13.9–27.4]	0.901	[0.77–0.96]	0.11	0.29
	Double *N* (MEP/M-max/RMS)		3.3	[2.5–4.7]	28.0	[20.9–42.2]	0.896	[0.76–0.96]	2.67	7.40
	Silent Period (ms)		8.2	[6.3–11.7]	8.2	[6.2–11.9]	0.820	[0.60–0.92]	8.27	22.93
	140% aMT									
	Single *N* (MEP/M-max)		0.11	[0.09–0.16]	15.5	[11.7–22.9]	0.922	[0.82–0.97]	0.10	0.27
	Double *N* (MEP/M-max/RMS)		2.7	[2.1–3.9]	22.5	[16.9–33.7]	0.926	[0.83–0.97]	2.33	6.45
	Silent period (ms)		7.9	[6.1–11.3]	7.0	[5.3–10.1]	0.835	[0.64–0.93]	8.53	23.64
	160% aMT									
	Single *N* (MEP/M-max)		0.09	[0.70–0.13]	14.0	[10.6–20.6]	0.941	[0.86–0.98]	0.09	0.25
	Double *N* (MEP/M-max/RMS)		3.03	[2.3–4.3]	27.1	[20.3–40.9]	0.898	[0.77–0.96]	2.76	7.65
	Silent period (ms)		8.5	[6.6–12.2]	6.5	[4.9–9.4]	0.920	[0.81–0.97]	9.76	27.05

TE, typical error; CI, confidence interval; CV, coefficient of variance; ICC, intra-class correlation; SEM, standard error of the measurement; MDC, minimal detectable change; MVC, maximal voluntary contraction; M-max, maximal compound action potential; RMS, root mean square; TMS, transcranial magnetic stimulation; aMT, active motor threshold; MEP, motor evoked potential.

### Reliability of silent period durations at different torque levels

**Figure 5:**
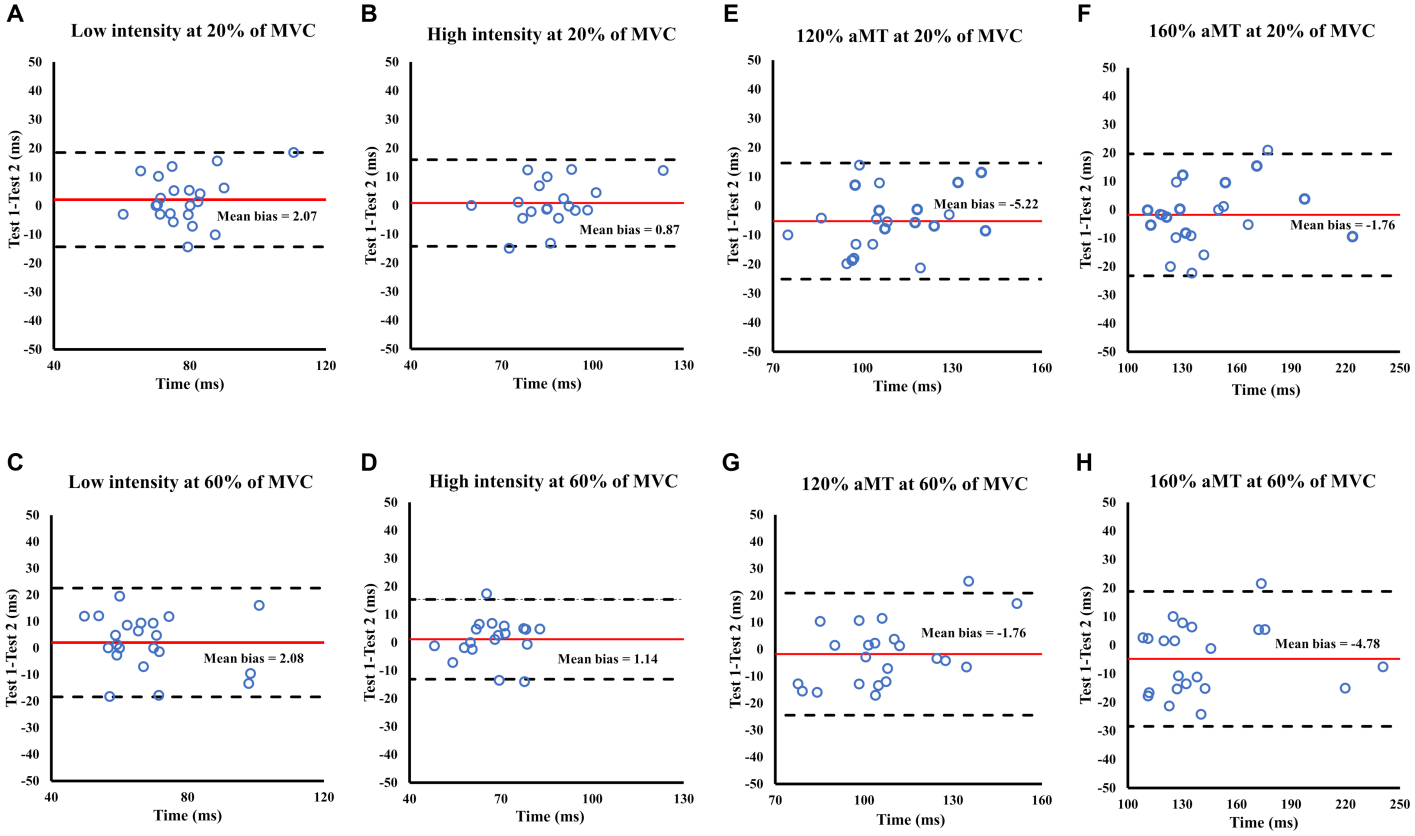
Bland Altman plots showing the level of agreement for LEP **(A–D)** and MEP **(E–H)** SPs during 20 and 60% of MVC between test sessions 1 and test 2. Each panel shows the differences as a function of the average of the two testing sessions indicating the mean bias (solid line) and the 95% limits of agreement (dashed lines).

SP showed a statistically significant difference at 120% aMT during 20% of MVC (*p* = 0.031) between test 1 and test 2, although the effect size was small (Hedges’ *g* = −0.28). No other condition showed any significant changes (Tables 2, 4). Moderate reliability was observed for low intensity LS at all contraction levels (Table 3). Excellent reliability was found for SP elicited by TMS at 160% aMT during 20 and 60% of MVC (ICC: 0.920 for both). Good reliability was found for high intensity LS and the rest of the TMS conditions and at any contraction level. CV for LS was between 7–12% and CV was between 6–8% for TMS. SEM for LS was between 13–22 and MDC was between 13–18. SEMs for TMS were between 7–10 and MDCs were 21–30. Bland–Altman plots showed good agreement between test 1 and test 2 regardless of the stimulation method, intensity, or contraction level (Figure 5).

## Discussion

The aim of the present study was to assess the reliability of MEP and LEP measures of cortico-spinal excitability during different submaximal contraction levels in the RF muscle of healthy adults. Our findings indicate that the use of MEP amplitudes normalized to M-max (Single *N*) and M-max/RMS (Double *N*) are reliable methods. In addition, Single *N* and Double *N* LEP amplitudes showed moderate reliability. Furthermore, MEP and LEP silent periods showed good-to-very good reliability. Moreover, small magnitude systematic bias demonstrated that MEPs, LEPs and their SPs are reliable tools to measure the cortico-spinal tract.

### Lumbar stimulation

This is the first study reporting LEP reliability during different submaximal contraction levels. LS can elicit a LEP in the target muscle, which represents the excitability of the motor-neuron (Škarabot et al., 2019b; Brownstein et al., 2021). Our results suggest moderate-to-good reliability of LEPs with 10 stimulations during different contraction levels and stimulator output intensities, independent of the normalization procedure. Despite the moderate reliability shown for some conditions (low intensity during 20% of MVC with Single N), these LEP values are within the range reported by previous MEP reliability studies investigating the lower limbs (ICC = 0.6–0.9) (Brownstein et al., 2018; Leung et al., 2018; Malcolm et al., 2021). Furthermore, the CV reported in the present study are lower than ones reported previously (e.g., 59% CV in Ansdell et al., 2020) for LEPs normalized to M-max. It is, however, important to mention that MDC was more than 100% in some conditions with Double *N*. Thus, LS could be used as a tool to understand spinal excitability in the lower limbs in various experiments typical in clinical neurophysiology or exercise science fields (e.g., pharmacological treatment, training intervention, fatigue intervention, balance perturbation).

### Transcranial magnetic stimulation reliability

MEPs elicited by TMS showed very good-to-excellent reliability (0.82–0.94), depending on the normalization procedure and the contraction level. In our study, reliability was good during 20% of MVC and excellent during 60% of MVC for Single N. Interestingly, Brownstein et al. (2018) reported ICC of 0.77–0.87 in RF during 10% of MVC, Temesi et al. (2017) reported ICC > 0.80 for MEPs elicited in RF during 20% of MVC but poor reliability (ICC = 0.59) was observed by Malcolm et al. (2021) who used maximal voluntary contractions. Malcolm et al. (2021) proposed some factors for their poor reliability at high contractions intensities, such as high variability of individual EMG between measurement sessions, motor units synchronization and signal cancelation, and intrinsic fluctuation in cortical and spinal excitability. Particularly during higher contraction intensities (>75% of MVC), the firing rate of motor-neurons increases, and with an increase in refractory period that could reduce the magnitude of the MEP (Todd et al., 2003; Goodall et al., 2009). MEPs increase their size with increasing contraction intensity seemingly up to 50–75% of MVC (Martin et al., 2006; Oya et al., 2008; Goodall et al., 2009; Škarabot et al., 2019b) depending on the muscle. In the present study, 60% of MVC was used. Therefore, the MEP reliability could have benefited from testing at this contraction intensity and the lower level of motor-neuron activation compared to the maximal voluntary contraction used in Malcolm et al. (2021). Consequently, these factors could have led to a reduction in variability and higher ICC values in the present study than those reported by Malcolm et al. (2021).

Furthermore, our results have similar or even lower CV than those reported during 10% (CV = 18–20%) (Brownstein et al., 2018; Leung et al., 2018) and 20% (CV = 21%) (O’Leary et al., 2015) of MVC. Moreover, the values for systematic bias reported in the present study suggest that TMS-elicited responses during 20 and 60% of MVC are a reliable tool to measure the cortico-spinal tract, for example, in studies expecting changes in the magnitude of 0.24–0.36 for Single *N* and 6–25 for Double *N* (Leung et al., 2018).

### Silent period reliability

The duration of the silent period can provide information about the inhibition at the cortical or spinal level (Inghilleri et al., 1993). Reliability of LS-elicited SP were moderate and good, which were slightly higher than the TMS-elicited SP reported by Di Virgilio et al. (2022). Furthermore, CV were similar to those reported previous (CV = 7–15%) (O’Leary et al., 2015; Leung et al., 2018; Di Virgilio et al., 2022). Moreover, reliability for TMS-elicited SP at different stimulation intensities and contraction level were good and excellent, respectively. Our results were in concordance with other reported in by other groups (O’Leary et al., 2015; Leung et al., 2018; Pagan et al., 2023). Furthermore, the CV of the TMS-elicited SP was within the ranges mentioned above. Therefore, our results suggest that SP could be used to understand inhibitory process at cortical and spinal segments by utilizing both TMS and LS concurrently.

### Strength and limitations

This study is the first to provide reliability statistics for two methods to assess cortico-spinal and spinal excitability during different submaximal contraction levels and stimulation intensities. Although previous studies have reported the reliability of MEPs at low submaximal contraction levels, this is the first that provides reliability for submaximal contraction levels higher than 20% of MVC (Temesi et al., 2017; Brownstein et al., 2018; Leung et al., 2018). Moreover, this is the first study reporting reliability of LEPs at different submaximal contraction levels. This study also provides reliability data of a normalization technique for MEPs and LEPs that aims to take into account the possible effect of EMG background activity on the induced responses (Sidhu et al., 2013; Škarabot et al., 2019c).

In terms of limitations, the number of stimuli might have been a possible factor for the LEPs moderate reliability. Although studies that have reported LEPs have used 10 stimuli, there is evidence from MEP reliability studies reporting that an increase in number of stimuli (>15) could improve reliability of MEPs (Bastani and Jaberzadeh, 2012; Cavaleri et al., 2017; Brownstein et al., 2018).

In conclusion, the results suggest that MEPs and LEPs are reliable tools to assess different segments of the cortico-spinal tract during different contraction levels and stimulator output intensities, independent of the normalization procedure. Thus, it may not be necessary to account for background EMG during TMS or LS stimulation when normalized to a valid maximal compound action potential. Furthermore, the TMS- and LS-elicited SP has also shown to be a reliable tool considered to reflect inhibitory processes at cortical and spinal levels.

## Data availability statement

The raw data supporting the conclusions of this article will be made available by the authors, without undue reservation.

## Ethics statement

The studies involving humans were approved by the Petteri Niemi University of Jyväskylä. The studies were conducted in accordance with the local legislation and institutional requirements. The participants provided their written informed consent to participate in this study.

## Author contributions

GG-G, JA, and SW: conceptualization. GG-G, ME, EH, PA, and GH: piloting and lab set up. GG-G, ME, EH, PA, and SW: data collection and data analysis. GG and SW: writing original draft. GG-G, PA, GH, JA, and SW: writing-reviewing-editing. All authors contributed to the article and approved the submitted version.

## Conflict of interest

The authors declare that the research was conducted in the absence of any commercial or financial relationships that could be construed as a potential conflict of interest.

## Publisher’s note

All claims expressed in this article are solely those of the authors and do not necessarily represent those of their affiliated organizations, or those of the publisher, the editors and the reviewers. Any product that may be evaluated in this article, or claim that may be made by its manufacturer, is not guaranteed or endorsed by the publisher.
