# Supplementary figures and images for: Test–retest reliability of cortico-spinal measurements in the rectus femoris at different contraction levels
Source: Front Neurosci. 2023 Oct 2;17:1239982. doi: 10.3389/fnins.2023.1239982 (PMC10577233; doi:10.3389/fnins.2023.1239982)

**A**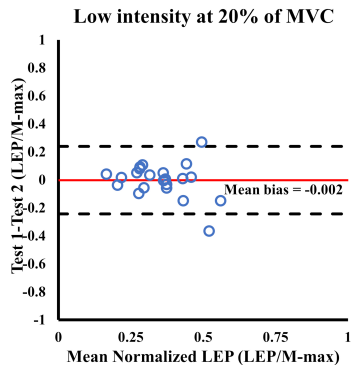**B**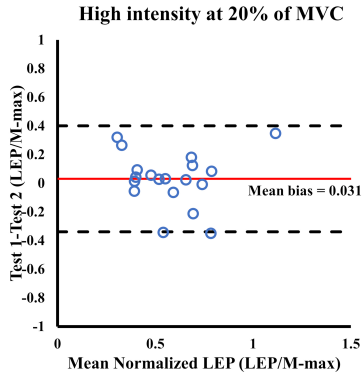**F**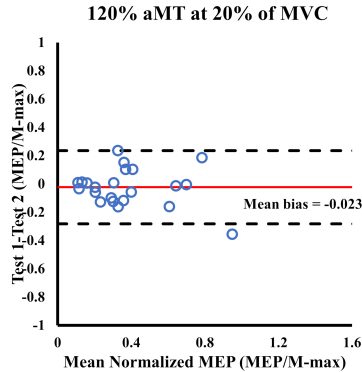**H**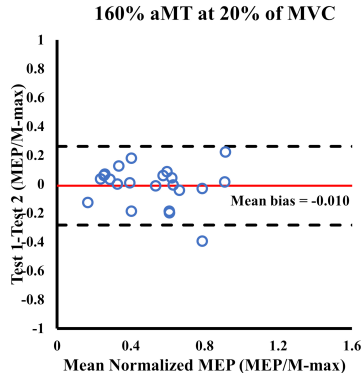**D**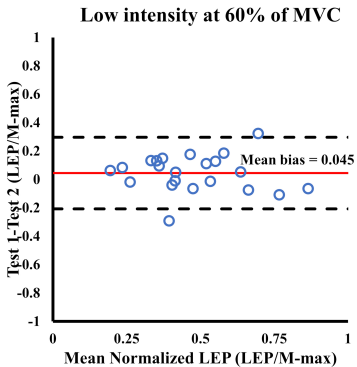**E**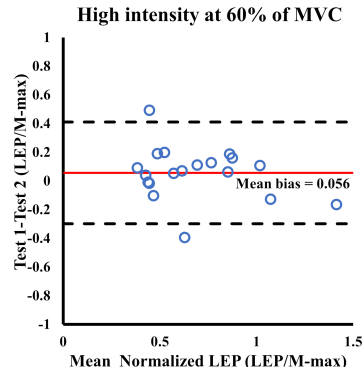**G**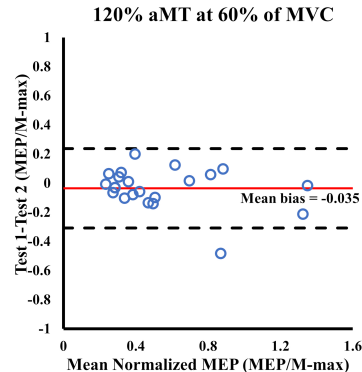**I**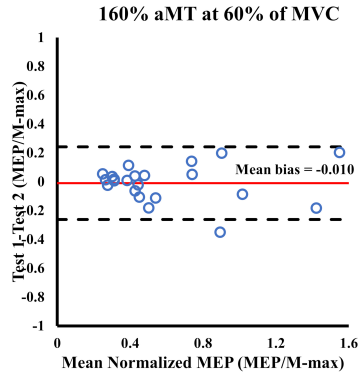

Supplement: Supplementary file 1 [file Data_Sheet_1.PDF]

**A**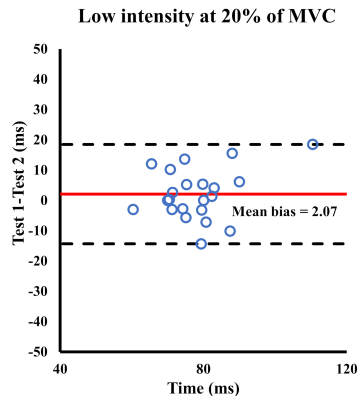**B**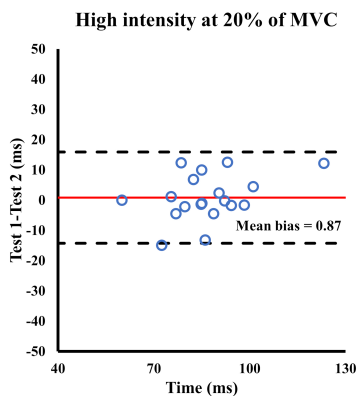**F**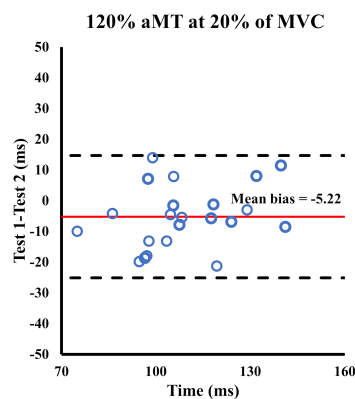**H**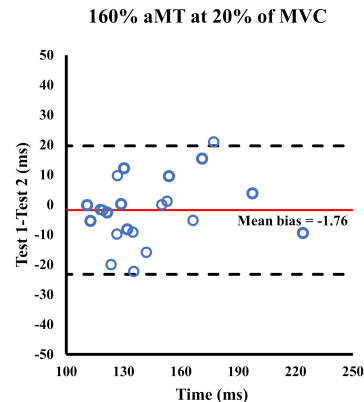**D**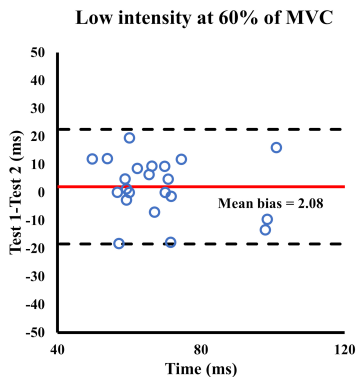**E**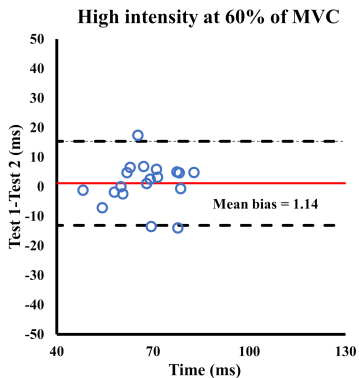**G**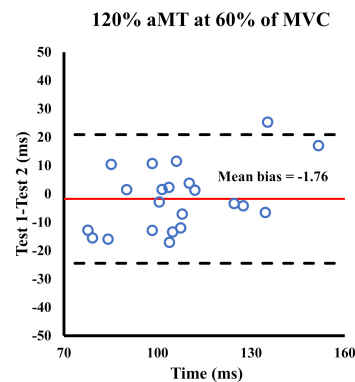**I**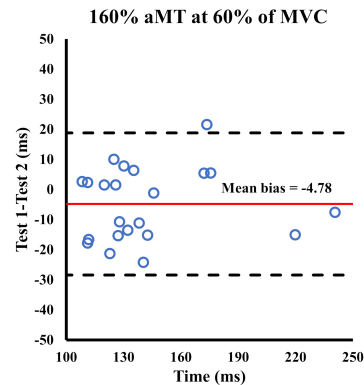

Supplement: Supplementary file 2 [file Data_Sheet_2.PDF]
